# Capturing the trends in hospital standardized mortality ratios for pneumonia: a retrospective observational study in Japan (2010 to 2018)

**DOI:** 10.1186/s12199-019-0842-4

**Published:** 2020-01-07

**Authors:** Rebeka Amin, Yosuke Hatakeyama, Takefumi Kitazawa, Kunichika Matsumoto, Shigeru Fujita, Kanako Seto, Tomonori Hasegawa

**Affiliations:** 1 Department of Social Medicine, Toho University Graduate School of Medicine, 5-21-16, Omori-nishi, Ota-ku, Tokyo, 143-8540 Japan; 2 Department of Social Medicine, Toho University School of Medicine, 5-21-16, Omori-nishi, Ota-ku, Tokyo, 143-8540 Japan; 3 Faculty of Health Sciences, Tokyo Kasei University, 2-15-1, Inariyama, Sayama-shi, Saitama, 350-1398 Japan

**Keywords:** Pneumonia, Japan, In-hospital mortality, Quality indicator, Health care/standards, Benchmarking

## Abstract

**Background:**

Pneumonia has a high human toll and a substantial economic burden in developed countries like Japan, where the crude mortality rate was 77.7 per 100,000 people in 2017. As this trend is going to continue with increasing number of the elderly multi-morbid population in Japan; monitoring performance over time is a social need to alleviate the disease burden. The study objective was to determine the characteristics of hospital standardized mortality ratios (HSMRs) for pneumonia in Japan from 2010 to 2018 to describe this trend.

**Methods:**

Data of the DPC (Diagnostic Procedures Combination) database were used, which is an administrative claims and discharge summary database for acute care in-patients in Japan. HSMRs were calculated using the actual and expected numbers of in-hospital deaths, the latter of which was calculated using logistic regression model, with a number of explanatory variables, e.g., age, sex, urgency of admission, mode of transportation, patient volume per month in each hospital, A-DROP score, and Charlson comorbidity index (CCI). We constructed two HSMR models: a single-year model, which included hospitals with > 10 in-patients per month and, a 9-year model, which included those hospitals with complete 9-year data. Predictive accuracy of the logistic models was assessed using c-index (area under receiver operating curve).

**Results:**

Total 230,372 patients were included for the analysis over the 9-year study period. Calculated HSMRs showed wide variation among hospitals. The proportion of hospitals with HSMR less than 100 increased from 36.4% in 2010 to 60.6% in 2018. Both models showed good predictive ability with a c-statistic of 0.762 for the 9-year model, and no less than 0.717 for the single-year model.

**Conclusion:**

This study denoted that HSMRs of pneumonia can be calculated using DPC data in Japan and revealed significant variations among hospitals with comparable case-mixes. Therefore, HSMR can be used as yet another measure to help improve quality of care over time if other indicators are examined in parallel and to get a clear picture of where hospitals excel and lack.

## Background

Pneumonia is the leading cause of morbidity (hospitalization) and mortality (in-patient deaths) associated with infectious diseases around the world and affects all age groups. Many developed countries, such as Japan, are now dealing with a super-aged society where multi-morbidity is a common scenario. That said, 132,629 people died from pneumonia, and it was 5th leading cause of death in Japan in 2017 [1]. Though many potent antibiotics and therapeutic strategies have been developed over several decades, 15 individuals still die every hour on an average from pneumonia in Japan [1].

In the past few decades, many efforts have been made to improve the quality of care. In-hospital mortality for common diseases shows considerable variation suggesting that there is potential for outcome improvement [2]. Hospital standardized mortality ratio (HSMR) is a representative risk-adjusted tool that measures mortality by taking account of factors known to affect the underlying risk of death [3].

HSMRs give hospital administrators and health providers a snapshot of a hospital’s performance at a given time and should be viewed in context with other indicators to help track progress over time. HSMRs have proven useful in many developed countries like the USA, Canada, Sweden, Wales, Australia, France, Singapore, and Hong Kong in identifying areas that can be changed to improve patient safety and quality of care [3–5].

Hospital administrative data have been used broadly for research and quality improvement efforts in recent years [6]. The Diagnostic Procedures Combination (DPC)/per-Diem payment system (PDPS) is a reimbursement method for acute care hospitals that was introduced in 2003, and DPC database is a national administrative claims and discharge abstract database for acute care in-patients in Japan [7, 8]. These data have been used in the design of health policies, disease management, and the analysis of healthcare processes and patient outcomes since its inception [9]. In Japan, several HSMR studies have been conducted [10–13] and the single-year HSMR model has already been created using administrative data for pneumonia [14]. Ian et al. reported that diagnosis-specific HSMR is potentially a more fruitful method for monitoring mortality over time, which allows for the earlier identification of care deficiencies [4]. However, studies regarding the trend of HSMRs for pneumonia are sparse in the international literature, and the trend for Japanese HSMRs for pneumonia has not yet been monitored and remains largely unknown.

The purpose of this study was to develop the calculation method of HSMR using the DPC data and determine the characteristics of HSMR for pneumonia in Japan from 2010 to 2018 to capture and describe the trend and to analyze which factors best explain this trend. To the very best of our knowledge this is the first large-scale study calculating HSMRs of pneumonia in Japan and revealing a 9-year trend using administrative data.

## Methods

For this retrospective observational study, HSMR method was used that was originally developed by Jarman in 1999 [3, 15, 16].

### Data

DPC data of the Medi-Target benchmarking project managed by the All Japan Hospital Association (AJHA) were used. The AJHA is one of the largest nation-wide hospital associations comprising of 2500 hospitals, which manages the administration of the Medi-Target project, a benchmark project using clinical indicators based on DPC data. Participation in the Medi-Target project was optional, and there were 182 participating hospitals in 2010, submitting about 500,000 claims data a year [8]. Details of the DPC database have been described elsewhere [12].

This study was based on the secondary analysis of the administrative claims data. Owing to the anonymous nature of the data, no IRB (Institutional Review Board) approval is necessary for this kind of study in Japan [17].

All hospital admissions with major diagnoses of pneumonia were identified from the DPC database for the year 2010 to 2018. ICD-10 code (J12-18, J69, B012, B052, B59) was used to determine the diagnoses. Hospitals that had ≤ 10 in-patients per month for pneumonia were excluded.

Patient and hospital-level data were collected as variables to analyze. Patient-level data included sex, age, urgency of admission (emergency or elective), mode of transportation (ambulance use), comorbidities on admission and during hospital stay, A-DROP score on admission, length of stay (LOS), operative status (surgery done or not), days of admission (weekend/ weekdays), referral status, and in-hospital death. A-DROP scoring system, developed by the Japanese Respiratory Society (JRS), is a modified version of CURB-65 (confusion, BUN > 7 mmol/L, respiratory rate ≥ 30/min, low blood pressure, and age ≥ 65 years) and has a higher level of discrimination than both CURB-65 and PSI (pneumonia severity index), with a reported c-statistic of 0.85 [18, 19]. It is a 6-point scale (0–5) that assesses the following parameters: (1) age (male ≥ 70 years, female ≥ 75 years), (2) dehydration (blood urea nitrogen (BUN) ≥ 210 mg/L), (3) respiratory failure (SaO_2_ ≤ 90% or PaO_2_ ≤ 60 mmHg), (4) orientation disturbance (confusion), and (5) low blood pressure (systolic blood pressure ≤ 90 mmHg). The following scale represents the four levels of pneumonia severity: (1) 0, mild; (2) 1–2, moderate; (3) 3, severe; and (4) 4–5, extremely severe [19, 20]. The A-DROP score was reported as follows for the purpose of analysis: A-DROP score 0 as 0, 1–2 as 1, 3 as 2, and 4–5 as 3.

Then, Charlson comorbidity Index (CCI; range 0–6), which is derived from secondary ICD-10 diagnoses codes, was calculated. The CCI is a weighted score based on the number and type of diagnoses reported in the hospital summary data [21, 22]. CCI was calculated based on Deyo’s modification [23, 24].

Hospital-level data included patient volume per month, presence of patient safety manager, and type of the hospital (teaching hospital or not).

Outcome variable was the overall in-hospital mortality and is referred to death that occurs at any point during the entire admission period.

### Calculation of HSMR

HSMR is defined as the ratio of the actual number of in-hospital deaths to the expected number of such deaths multiplied by 100 [25].

$$ \mathrm{HSMR}=\left(\frac{\mathrm{Observed}\ \mathrm{number}\ \mathrm{of}\ \mathrm{deaths}}{\mathrm{Expected}\ \mathrm{number}\ \mathrm{of}\ \mathrm{deaths}}\right)\times 100 $$

The observed number of deaths is the sum of the actual number of deaths in that hospital. The expected number of death for a hospital is based on the sum of the probabilities of in-hospital deaths. An HSMR above or below 100 indicates that the mortality rate is higher or lower, respectively, than the overall average.

First, a multivariable logistic regression model was constructed to predict the chance of in-hospital death for each patient with patient-level and hospital-level factors. Logistic regression analyses were performed to calculate the intercept of covariates. Covariates for case mix adjustment are sex, age, urgency of admission, mode of transportation, patient volume per month in each hospital, A-DROP score, and CCI. All variables are categorical except age, CCI, and patient volume, which are continuous variable. All independent variables were entered into the equation in one step (forced-entry method). Coefficients derived from logistic regression models were used to calculate the probability of in-hospital death. Then, summation of the predicted probabilities of deaths (ranging from 0 to 1) gives the total expected number of in-hospital deaths in that hospital. Ratio between the expected numbers of in-hospital deaths and the actual number of deaths gives the standardized mortality ratio for that hospital of interest.

We constructed two models of HSMR. First one included the hospitals, which had >10 pneumonic patients per month; therefore, number of hospitals are different for each year. This is called the single-year model. The second is a 9-year model, which was created to assess the trend of HSMRs over time, by including hospitals which had complete data for 9 years. Fitting data from all 9 years into one model allowed us to make valid comparisons over time.

### Statistical analysis

Following variables were examined for their association with in-hospital mortality: sex, age, urgency of admission, mode of transportation, LOS, operative status, A-DROP score at admission, and CCI. Patient characteristics of the dead and surviving groups were compared using either Chi-square tests for categorical variables, *t* tests for normally distributed continuous variables, and non-parametric tests (Mann-Whitney *U* test) for non-normally distributed continuous variables. Continuous variables were summarized with the use of descriptive statistics (mean ± standard deviation for normally distributed values, and the median (25 percentile, 75 percentile) for non-normally distributed ones), and categorical variables were summarized as frequencies and proportions. *P* values < 0.05 were considered statistically significant.

Predictive accuracy of the logistic model was assessed using c-index. The c-index is derived by calculating the proportion of concordant pairs and is equivalent to the area under a receiver operating curve. A c-index value of 0.5 suggests that the model is no better than random chance in predicting death, whereas a value 1.0 indicates perfect discrimination.

The association between the HSMRs for each year was evaluated using the Spearman’s correlation coefficient. A multivariable logistic regression was modeled to analyze the association between HSMRs and any contributing factor (e.g., percentage of referred patient, percentage of weekend admission, type of hospital, and presence of patient safety manager). All contributing factors were counted as independent variables and entered into the equation in one step (forced-entry method). HSMRs were classified into one of two groups (HSMR ≤ 100 and HSMR > 100) and counted as dependent variable. Within the targeted 9 years, some hospitals changed their affiliation status (academic/not academic), and newly appointed a patient safety manager. Therefore, we used these hospital variables in the 9-year model as the proportion of patients admitted for the situation (status of the hospital at the time of admission).

In the 9-year model, 95% confidence intervals (CIs) of the HSMRs for 2010–2018 were calculated using Byar’s approximation. All statistical analyses were performed by using the Statistical Package for Social Science (SPSS), version 17.0.0.

## Results

### Characteristics of study population

After exclusions, the overall sample size for the analysis was 230,372 patients over the 9-year study period (January 2010 to December 2018) with major diagnosis of pneumonia. Table 1 shows the demographics of the overall sample and Additional file 1: Table S1 shows the demographics in single-year models. Of the 230,372 patients included in the development dataset, 90.3% were discharged alive, 5.6% patients underwent surgery, 22.3% patients were admitted on a weekend, and 54.1% patients had a referral letter. Essential hypertension was the single-most commonly occurring (11,205 patients) comorbidity among pneumonia patients at admission. Other associated comorbidities at admission included volume depletion (9071), type 2 diabetes mellitus (8796), acute respiratory failure (8569) and asthma (7574). The top five comorbidities after admission were muscle atrophy (19,392), aphasia and dysphagia (4771), and constipation (3544), insomnia (2529), and malignant neoplasm of bronchus and lung (2274). Among all, 23.3% patients were ≤ 20 years of age and 71.6% patients were ≥ 60 years of age.

**Table 1:** Patient demographics (9-year)*

Characteristics		2010–2018 (*n* = 230,372)		
		Alive	Dead	*P* value
Demographic features				
Age (years)	Mean ± SD	60.0 ± 34.9	85.1 ± 8.9	< 0.001^†^
	Median (IQR)	77 (22, 86)	86 (81, 91)^†^	< 0.001^††^
Sex (male)		113,530 (54.6)	13,297 (59.6)	< 0.001^¶^
Comorbidity				
CCI	Mean ± SD	3.4 ± 2.3	5.2 ± 1.6	< 0.001^†^
	Median (IQR)	4 (1, 5)	5 (4, 6)^†^	< 0.001^††^
Admission features				
LOS (days)	Mean ± SD	18.1 ± 22.6	37.3 ± 67.3	< 0.001^†^
	Median (IQR)	11 (7, 21)	21 (9, 44)^†^	< 0.001^††^
Emergency admission		204,057 (97.9)	21,208 (97.4)	< 0.001^¶^
Ambulance usage		52,869 (25.4)	10,566 (47.4)	< 0.001^¶^
Severity status				
A-DROP score 0		109,504 (52.6)	8693 (38.9)	< 0.001^¶^
A-DROP score 1		68,681 (33.0)	4927 (22.1)	
A-DROP score 2		17,386 (8.4)	3686 (16.5)	
A-DROP score 3		12,482 (6.0)	5013 (22.5)	

^*^Values in parentheses are %

^†^*T* test

^††^Mann-Whitney *U* test

^¶^Chi-square test

*CCI* Charlson comorbidity index, *LOS* length of stay

Patient characteristics of each year in the single-year model showed a similar trend to the 9-year model, which increases the robustness of the results.

### Hospital characteristics

A total of 168 hospitals were included in the single-year model and 33 were included in the 9-year model. Among these, 75.6% are teaching hospital and 67.9% have a patient safety manager.

### In-hospital mortality (single-year model)

**Fig. 1:**
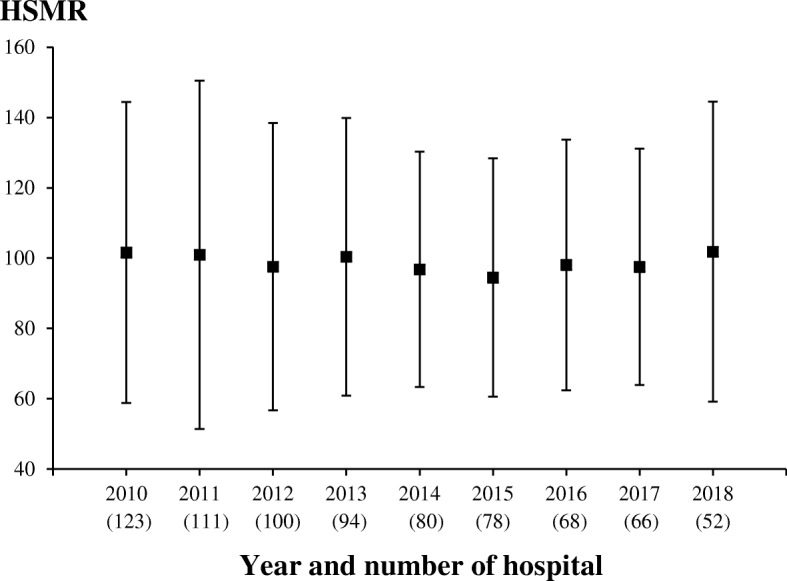
Mean and SD of the HSMR for pneumonia for each year. HSMR hospital standardized mortality ratio

The HSMR widely varied across the hospitals included in this study. Figure 1 shows the variation of mean and standard deviation (SD) of each year’s HSMR. The HSMRs ranged from 17 to 311% in 2010, 25 to 317% in 2011, 21 to 249% in 2012, 23 to 219% in 2013, 37 to 196% in 2014, 29 to 193% in 2015, 36 to 218% in 2016, 43 to 219% in 2017, and 34 to 239% in 2018. Of the hospitals evaluated, 62 (50.4%), 48 (43.2%), 46 (46.0%), 43 (45.7%), 37 (46.3%), 33 (42.3%), 25 (36.8%), 26 (40.0%), and 23 (44.2%) had higher mortality rate than expected in 2010, 2011, 2012, 2013, 2014, 2015, 2016, 2017, and 2018, respectively.

### Statistical analysis

c-index showed strong a predictive ability for both models, i.e., 0.762 for 9-year model, and 0.823, 0.847, 0.770, 0.734, 0.730, 737, 0.745, 0.727, and 0.717 in 2010, 2011, 2012, 2013, 2014, 2015, 2016, 2017, and 2018, respectively, for single-year model. Results are shown in the supporting figure (Additional file 2: Figure S1).

Table 1 and Additional file 1: Table S1 compare the patient characteristics with mortality for statistical significance. All the analyzed variables showed a statistically significant relationship with mortality (*P* value < 0.05).

To check the change in each year’s HSMR, the Spearman’s non-parametric correlation coefficient between each consecutive year was calculated. The correlation analysis revealed a significant, positive relationship between the changes in the HSMR of each consecutive year (Table 2).

**Table 2:** Relationship between the HSMRs of each consecutive year (single-year model)

Month and year	*N*	*R*	*P*
Jan. 2010–Dec. 2011	93	0.720	< 0.001
Jan. 2011–Dec. 2012	95	0.616	< 0.001
Jan. 2012–Dec. 2013	85	0.687	< 0.001
Jan. 2013–Dec. 2014	73	0.761	< 0.001
Jan. 2014–Dec. 2015	70	0.760	< 0.001
Jan. 2015–Dec. 2016	63	0.768	< 0.001
Jan. 2016–Dec. 2017	62	0.762	< 0.001
Jan. 2017–Dec. 2018	50	0.649	< 0.001

*N* number of hospitals, *R* correlation coefficient (Spearman’s non-parametric correlation), *P* two-tailed significance

Positive correlation coefficient means that hospitals with lower/higher HSMRs are likely to get same results in the following year

To determine those factors influencing the HSMR, variables were further examined by logistic regression analysis using the 9-year model. Table 3 shows the odds ratio from these analyses. Only the presence of a patient safety manager had a statistically significant influence on HSMR (OR, 0.97; 95% CI, 0.95-1.00; *P* value, 0.03).

**Table 3:** Factors influencing HSMR in pneumonia patients (9-year model, 2010–2018, *n* = 33)

Contributing factors	HSMRs (HSMR > 100)*		
	Odds ratio	95% CI	*P* value
% of hospital with patient safety manager^†^	0.97	0.95–1.00	0.03
% of academic hospital^††^	0.98	0.96–1.00	0.10
% of weekend admission	1.14	0.91–1.42	0.27
% of patients referred^¶^	1.04	0.97–1.11	0.28
Negelkerke *R*^2^		0.36	

^†^Percentage of patients admitted into a hospital with patient safety manager

^††^Percentage of patients admitted into a hospital with academic status

^¶^Percentage of patients who had a referral letter on admission

^*^HSMRs were classified into one of two groups, HSMR ≤ 100 and HSMR > 100

*CI* Confidence interval

### Nine-year trend

**Fig. 2:**
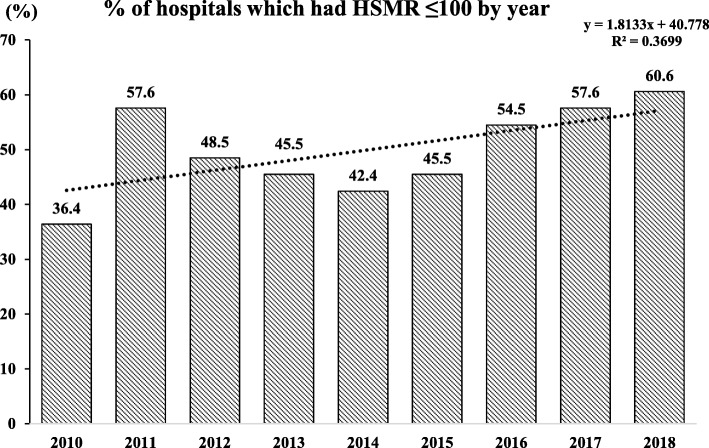
Trend changes in the percentage of hospitals with HSMR ≤ 100 from 2010 to 2018. Nine-year model, *n* = 33. HSMR hospital standardized mortality ratio

For this time series analysis, a total of 33 hospitals were included. The percentage of hospitals with mortality lower than the expected rates increased from 36.4% in 2010 to 60.6% in 2018, indicating a positive linear relationship (Fig. 2). The percentage change of HSMR in this 9-year period ranged from − 0.11 to 0.04%.

**Fig. 3:**
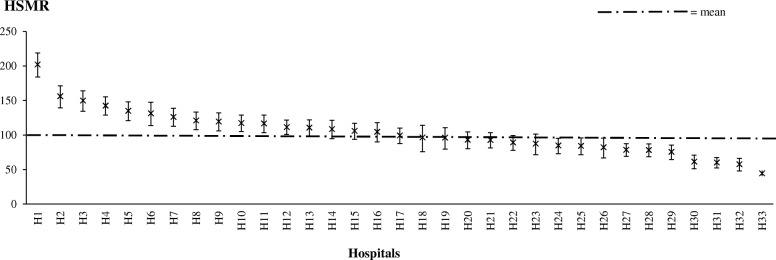
Caterpillar plot of the HSMR for pneumonia (9-year model, 2010–2018, *n* = 33) for each hospital. The HSMR is graphed as the upper and lower 95% confidence limits. HSMR hospital standardized mortality ratio

Mean and 95% CIs for each hospital have been graphed in Fig. 3. While the lower limit varied from 36.1 to 185.9, the upper limit varied from 54.2 to 219.4. The mean HSMR was 102.0**.**

## Discussions

This study denoted that HSMRs can be calculated using DPC data in Japan. Findings of this study have revealed that the HSMRs of pneumonia varied significantly among hospitals in Japan with comparable case-mixes. The hospital with the highest HSMR was 4.9–18.8 times higher than that of the lowest HSMR hospital. Approximately 5.0%-11.7% hospitals had subpar HSMR (i.e., > 150), and 5.9–10.0% hospitals had good HSMR (i.e., <50).

After adjustment for case-mixes, some hospitals were found to have a higher mortality rate. Overall, 9.7% died among the total study population. Population aging is one of the most dramatic trends in the world today, and Japan is considered to be a forerunner of an ever-aging world [26, 27]. In this era of aging, treating multi-morbid, and sometimes immune-deficient aged patients with chronic conditions is becoming more difficult, as is choosing an appropriate antibiotic to treat them. Though many potent antibiotics produce similar beneficial outcomes in all age groups, an increasing age is generally associated with more severe symptoms and worse functional outcomes. On the other hand, dealing with emerging multi-drug resistant bacteria, and viral pneumonia is equally difficult.

The most remarkable finding of this study is that of the results of correlation analysis, which portrays that those hospitals with lower HSMRs are likely to produce the same results in the following year. On the other hand, institutions with higher HSMRs are also expected to produce the same results the following year. This needs to be analyzed in-depth.

The 9-year HSMR model reveals the tapering trend of the HSMRs, which is consistent with other studies in Japan and other countries [14, 26]. Release of management guidelines by JRS in 2000 [20], and adherence to the JRS guidelines, substantial progress in therapeutic options, and availability of potent antibiotics might have influenced the general reduction in mortality from pneumonia. Also, introduction of the PCV7 (7-valent pneumococcal conjugate) vaccine has decreased proportion of pneumococcal pneumonia in both adults and children [28].

Measuring and assessing hospital quality and determining all of the contributing factors that affect changes to the HSMR is not only demanding but also challenging. Thus, apart from quality issues, in-hospital mortality might be influenced by many factors, including admission and discharge practices. It is noteworthy that part of the reduction in HSMRs may be due to chance or better coding, different discharge policies, referral of more complicated patients to other hospitals. Each of these factors may influence the calculation of HSMRs.

Another notable finding of this study is the significant relationship between the presence of a patient safety manager and HSMRs, which indicates that the presence of a patient safety manager might reduce the HSMR. This is a call for a thorough evaluation, as this finding matches with a previous study, and might open up a new platform to work on and new strategies that can be implemented [29]. However, this study was unable to find any significant relationship with HSMRs and a weekend admission, academic status of the hospital or the referral of patients, though another study has found a weekend effect for pneumonia [9].

### Strengths and limitations

This study possesses some strengths. Firstly, it included a large sample size and several variables from an administrative database. Secondly, a risk-adjusted model was established and internally validated, which demonstrated the variation in pneumonia mortality among hospitals over a period of 9 years. Thirdly, though only a small number of hospitals was included, we assume that the 9-year model is free from miscoding, which increases the credibility of the data. Lastly, patients from throughout the year were included in the study, so seasonal variation of pneumonia could not influence our results.

We have to admit a few limitations of this study. Firstly, as this is a secondary analysis of administrative data, the variables reported by hospitals were not controlled. Secondly, tests for statistical significance were not done for time trend. As such, cautious interpretation of results is recommended. Additionally, the DPC database does not provide a detailed medication history for patients. Thus, adherence to the JRS guidelines, for example, “commencement of antibiotic therapy within 4 hours of admission,” was not examined. Furthermore, the samples were obtained from hospitals that voluntarily submitted data, so the study population might not be representative of the whole population in Japan, and the overall mortality rate may be underestimated. Another concern is how to deal with any missing data. However, as percentage of missing data was very low (i.e., 0.1–0.3%), we believe that could not affect the result. Moreover, no hospital-level factors were analyzed besides their academic status and the presence of a patient safety manager. Factors such as the bed-to-physician or bed-to-nurse ratio, urban/rural location and presence of infectious disease specialists/ respiratory specialists might explain some of the observed differences among facilities [30–32]. It is worth mentioning that many hospitals have changed their discharge policies to shorten the LOS as a part of the health sector reform encouraged by the Ministry of Health, Labour and Welfare, Japan [33]. If patients were discharged to community services/long-term care facilities and died there, then they were not counted as a death in the calculation of HSMRs [34]. Each of these limitations might act as a potential confounder; therefore, any future analysis requires measures to overcome these limitations, which would result in more precise evaluations.

Of note, a number of arguments have been made saying that HSMR is flawed as a tool of quality indicator and cannot measure patient safety [4, 35]. We accept that hospital care is complicated and is dependent on many factors, not all of which are reflected by the HSMR. This is why various indicators must be examined in order to obtain a clear picture of hospital performance; where they excel and where they lack.

Strategies have been implemented in hospitals that have embraced this approach in the USA and the UK [5, 36–38], and HSMR has been reported publicly in Canada and the Netherlands [3, 4, 15]. As a tool to help health professionals follow trends in mortality rate, the HSMR can be useful as yet another measure for designing targeted interventions to help improve quality of care and patients’ experience over time.

## Conclusions

The results of this study have shown that it is possible to use data from the DPC to compute the HSMR and that the HSMRs of pneumonia varied significantly among hospitals with comparable case-mixes. Risk-adjusted, internally validated models might contribute to developing more appropriate benchmarking systems for inter-hospital comparisons. However, measuring and assessing hospital quality is not only demanding but also a difficult task; thus, these results should be interpreted with great caution.

## Supplementary information

**Additional file 1: Table S1.** Patient demographics (single-year)*.

**Additional file 2: Figure S1.** Predictive ability of the models (single-year model and 9-year model).

## Data Availability

The data that support the findings of this study are available from the All Japan Hospital Association but restrictions apply to the availability of these data, which were used under license for the current study, and so are not publicly available. Data are however available from the authors upon reasonable request and with permission of the All Japan Hospital Association.

## Abbreviations

CCI: Charlson comorbidity index
CI: Confidence intervals
DPC: Diagnostic Procedures Combination
HSMRs: Hospital standardized mortality ratios
ICD: International Classification of Diseases
IRB: Institutional Review Board
JRS: Japanese Respiratory Society
LOS: Length of stay
PCV7: 7-valent pneumococcal conjugate vaccine
PDPS: Per-Diem Payment System
PSI: Pneumonia severity index
SD: Standard deviation
