# Supplementary figures and images for: Capturing the trends in hospital standardized mortality ratios for pneumonia: a retrospective observational study in Japan (2010 to 2018)
Source: Environ Health Prev Med. 2020 Jan 7;25:2. doi: 10.1186/s12199-019-0842-4 (PMC6947928; doi:10.1186/s12199-019-0842-4)

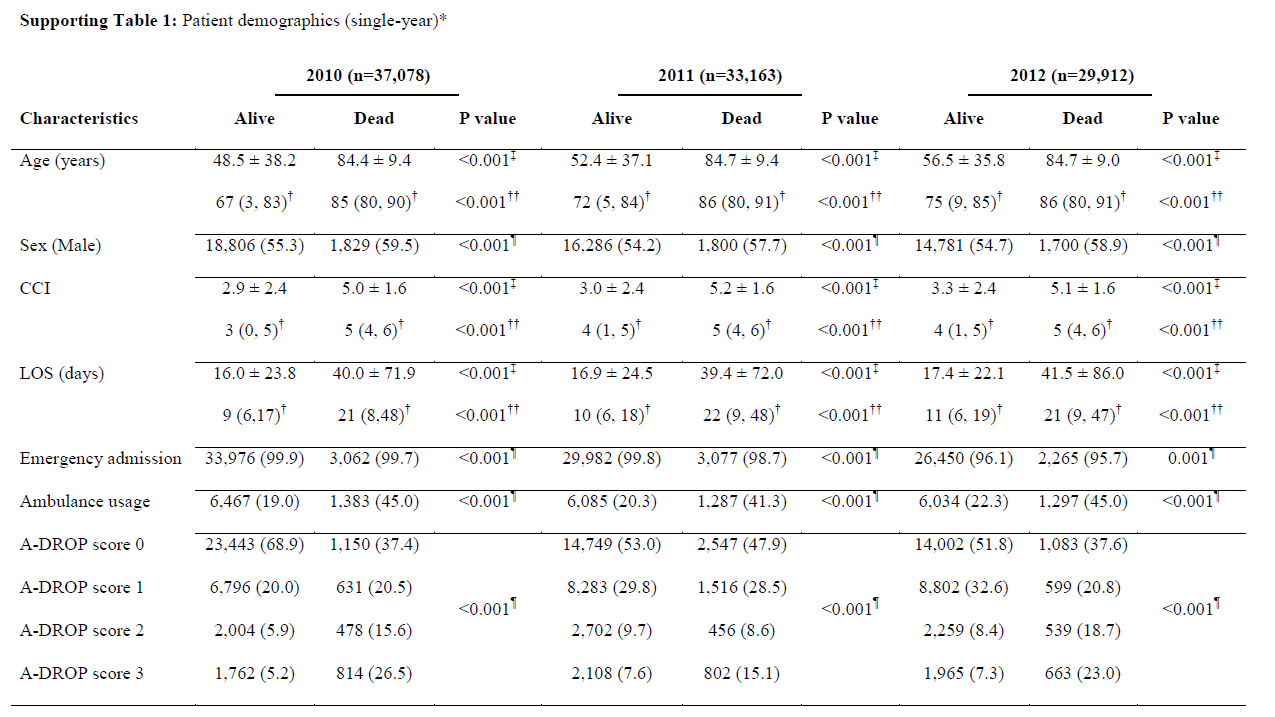


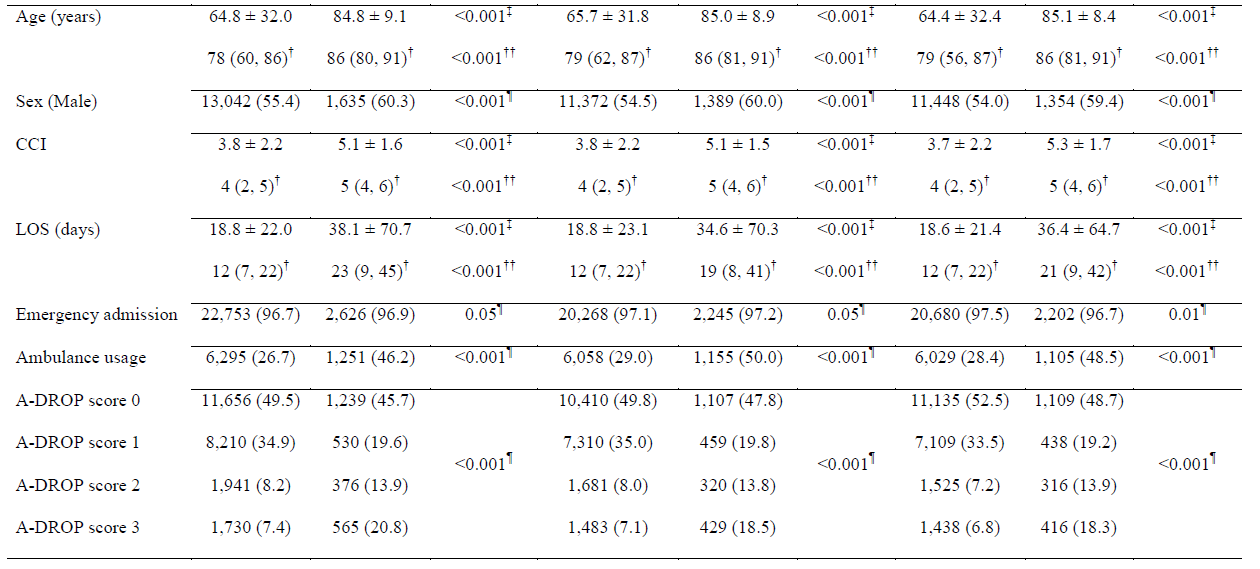


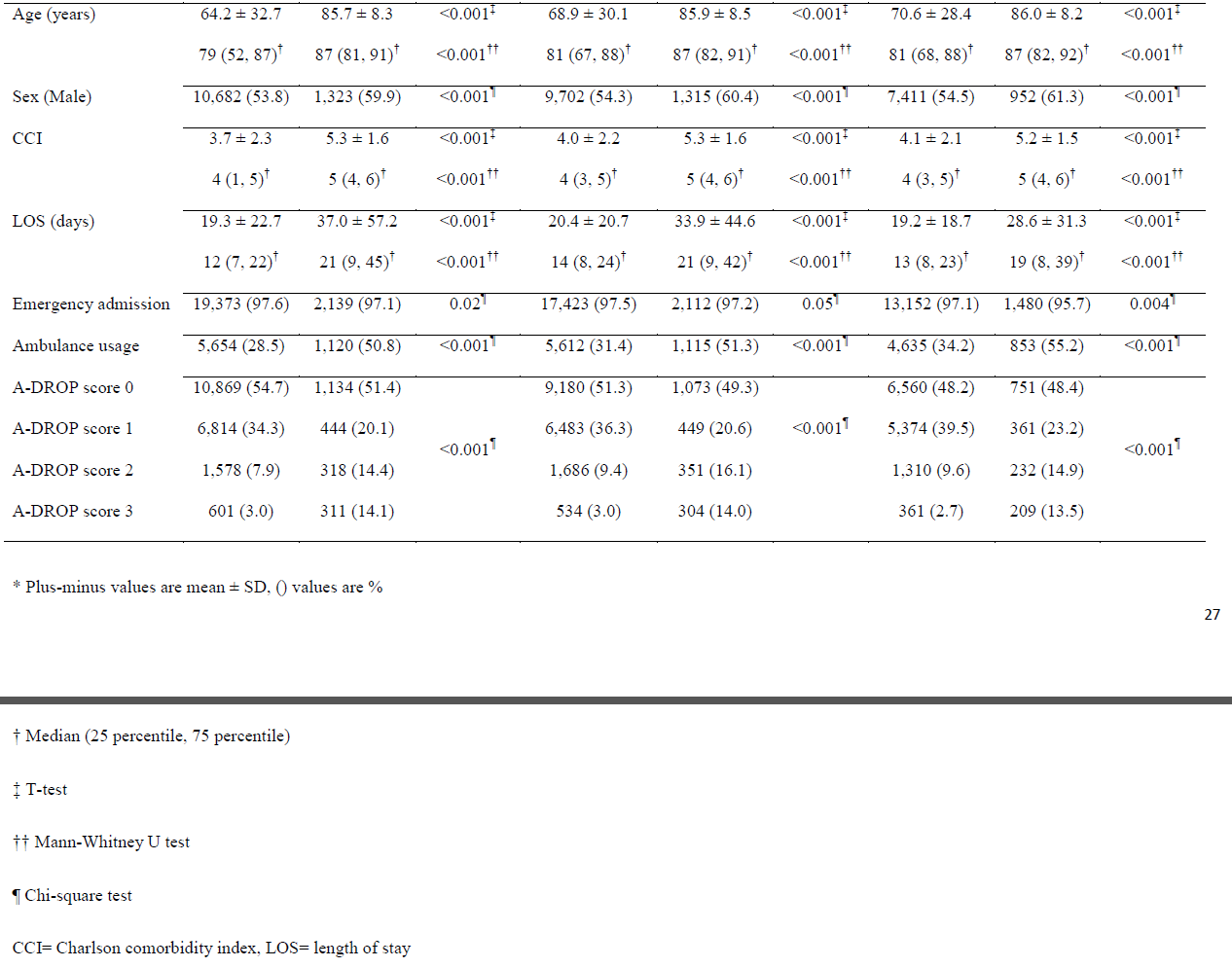

Supplement: Supplementary file 1 — Additional file 1: Table S1. Patient demographics (single-year)*. [file 12199_2019_842_MOESM1_ESM.docx]

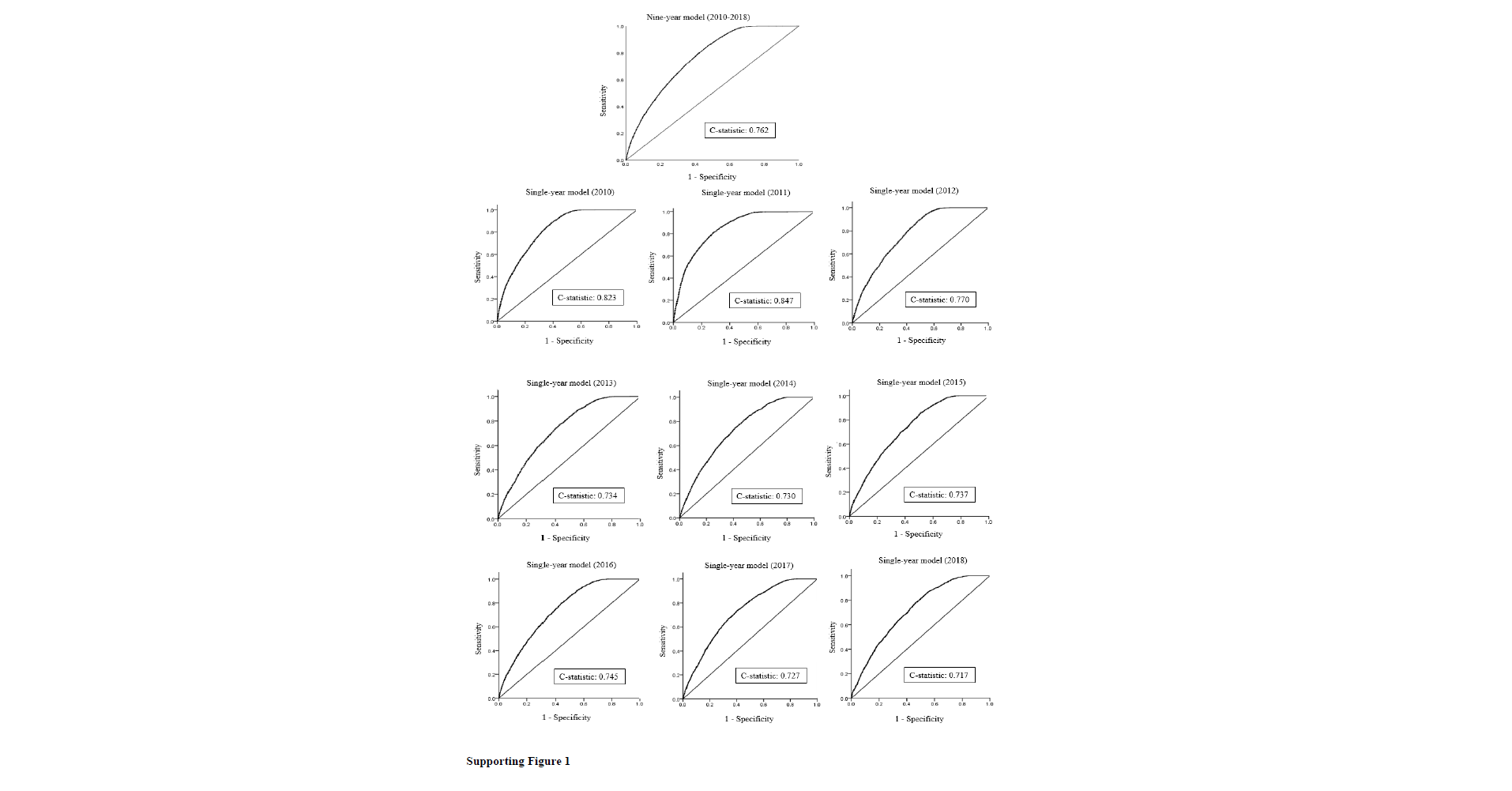

Supplement: Supplementary file 2 — Additional file 2: Figure S1. Predictive ability of the models (single-year model and 9-year model). [file 12199_2019_842_MOESM2_ESM.png]
